# Measurement properties of the Dizziness Handicap Inventory by cross-sectional and longitudinal designs

**DOI:** 10.1186/1477-7525-7-101

**Published:** 2009-12-21

**Authors:** Anne-Lise Tamber, Kjersti T Wilhelmsen, Liv Inger Strand

**Affiliations:** 1 Faculty of Health Sciences, Oslo University College, Norway; 2 Institute of General Practice and Community Medicine, Faculty of Medicine, University of Oslo, Norway; 3 Department of Physiotherapy, Bergen University College, Norway; 4 National Centre of Vestibular Disorders, Department of Otorhinolaryngology/Head and Neck Surgery, Haukeland University Hospital, Bergen, Norway; 5 Department of Public Health and Primary Health Care, Section for Physiotherapy Science, University of Bergen, Norway

## Abstract

**Background:**

The impact of dizziness on quality of life is often assessed by the Dizziness Handicap Inventory (DHI), which is used as a discriminate and evaluative measure. The aim of the present study was to examine reliability and validity of a translated Norwegian version (DHI-N), also examining responsiveness to important change in the construct being measured.

**Methods:**

Two samples (n = 92 and n = 27) included participants with dizziness of mainly vestibular origin. A cross-sectional design was used to examine the factor structure (exploratory factor analysis), internal consistency (Cronbach's α), concurrent validity (Pearson's product moment correlation r), and discriminate ability (ROC curve analysis). Longitudinal designs were used to examine test-retest reliability (intraclass correlation coefficient (ICC) statistics, smallest detectable difference (SDD)), and responsiveness (Pearson's product moment correlation, ROC curve analysis; area under the ROC curve (AUC), and minimally important change (MIC)). The DHI scores range from 0 to 100.

**Results:**

Factor analysis revealed a different factor structure than the original DHI, resulting in dismissal of subscale scores in the DHI-N. Acceptable internal consistency was found for the total scale (α = 0.95). Concurrent correlations between the DHI-N and other related measures were moderate to high, highest with Vertigo Symptom Scale-short form-Norwegian version (r = 0.69), and lowest with preferred gait (r = - 0.36). The DHI-N demonstrated excellent ability to discriminate between participants with and without 'disability', AUC being 0.89 and best cut-off point = 29 points. Satisfactory test-retest reliability was demonstrated, and the change for an individual should be ≥ 20 DHI-N points to exceed measurement error (SDD). Correlations between change scores of DHI-N and other self-report measures of functional health and symptoms were high (r = 0.50 - 0.57). Responsiveness of the DHI-N was excellent, AUC = 0.83, discriminating between self-perceived 'improved' versus 'unchanged' participants. The MIC was identified as 11 DHI-N points.

**Conclusions:**

The DHI-N total scale demonstrated satisfactory measurement properties. This is the first study that has addressed and demonstrated responsiveness to important change of the DHI, and provided values of SDD and MIC to help interpret change scores.

## Background

The Dizziness Handicap Inventory (DHI) is used in clinical work and in research to assess the impact of dizziness on quality of life. The self-report questionnaire was originally designed to quantify the handicapping effect of dizziness imposed by vestibular system disease [1], but has also been used for persons with dizziness of other origins [2-5]. The original American version has been translated and adapted to several languages and cultures, like Swedish [6], Chinese [7], and Dutch [8]. Translation of the DHI has also been demanded by clinicians and researchers in Norway.

Items included in the DHI were originally derived from case histories of patients with dizziness, and the measure was further examined in several studies involving patients seen for vestibulometric testing [1]. The DHI contains 25 items, and a total score (0-100 points) is obtained by summing ordinal scale responses, higher scores indicating more severe handicap. The scale was developed to capture various sub-domains of self-perceived handicap and comprises 7 physical, 9 functional, and 9 emotional questions [1]. Later studies of the underlying factor structure of the DHI failed to support the empirically developed sub-domains [9-11], which was also adressed in a recent review article [12].

High internal consistency has been demonstrated for the total scale as well as for the subscales [1]. Validity of the DHI was indicated as higher scores were associated with higher frequency of dizziness [1] and with greater functional impairments [13]. Concurrent validity has been examined in several studies, presenting variable results [14-16]. Satisfactory test-retest reliability has been demonstrated for the total scale as well as for the subscales, and a change in the DHI total score should decrease by at least 18-points in individual patients to be called a true change [1]. The ability of the DHI to measure meaningful or clinically important change, has scarcely been examined [12], and variable results regarding the ability of the DHI to discriminate between treatment and control groups, have been found in randomized controlled trials [17-25]. The ability to detect real change in the concept being measured, or the ability to distinguish between participants who have and have not changed an important amount [26,27], have not been reported.

Valuable information can be derived in the clinic from tools assessing self-perceived consequences of dizziness, presupposed satisfactory measurement properties. After translating the DHI into Norwegian, the aim of the present study was to examine reliability and validity of the translated version, which was to be used as a descriptive and evaluative measure. Responsiveness to important change in the construct being measured was included, as this has not been reported in the original DHI. Regarding construct validity and responsiveness, the hypotheses of correlations between scores of the DHI Norwegian version and other related measures, are defined in Methods (Statistical analysis).

## Methods

### Translation

The translation followed international guidelines through a process of reviews and modification [28,29]. Permission to translate the DHI into Norwegian was granted by Gary P. Jacobson, one of the test developers [1]. Translations from American to Norwegian were made separately by two physiotherapists familiar with dizzy patients and knowledgeable in American and English. The translated versions were discussed, and adjusted to obtain consensus and close equivalence with the original version [29]. Back-translation was performed by a bilingual person with Norwegian and English at a professional academic level, and with English as a native language. The original and the back-translated English versions were compared by the three translators, and if discrepancies were found, the Norwegian version was adjusted to optimize conceptual overlap [30]. The translated version was pilot tested on a few Norwegian speaking patients with dizziness (n = 4), and no particular problems were met regarding answering the questions. The DHI in a Norwegian version (DHI-N) is presented in Additional file 1, the sequence of rating alternatives in line with Jacobson & Newman [31]: Yes = 4, Sometimes = 2, No = 0.

### Design

A cross-sectional design was used to examine internal consistency and aspects of validity, and longitudinal designs were used to examine test-retest reliability and responsiveness.

### Participants

#### Sample 1

Potential participants with complaints of dizziness from the Oslo-Akershus region were recruited from General Practice, ENT-specialists and the National Insurance Administration (NIA 2003-2004). They received written information about the project during the doctoral visit, and/or by mail from the NIA, if registered with sick leave because of dizziness during the last year. Inclusion criteria were dizziness, age range 20-65 years, ability to read and understand Norwegian language, and living in the Oslo-Akershus region. Exclusion criteria were dizziness because of cardio-vascular disease, neurological or other severe system diseases, and not being able to answer the questionnaires or go through physical tests. Of the 112 individuals who volunteered for the study, 14 did not meet the inclusion criteria, i.e. 98 participants were included.

#### Sample 2

Patients between the ages of 18-70 years, examined in a balance clinic at Haukeland University Hospital, Bergen during the period of 2003-2005 were included provided that their medical examination, which included standard laboratory tests, suggested uncompensated vestibular function as a consequence of vestibular neuronitis. Exclusion criteria were evidence of central vestibular disorder or progressive vestibular pathology, including Ménière's disease, genetic hearing loss and/or neurological/musculoskeletal/visual/psychiatric disorders. Thirty-six patients were included, 32 of these were asked to participate in the reliability study.

The study was performed in accordance with the Helsinki Declaration. Written informed consent was obtained from all participants. The participants in sample 1 were part of a larger study approved by the Regional Committee for Medical Research Ethics, Health Region South, Norway. The participants in sample 2 were part of a larger study approved by the Regional Committee for Medical Research Ethics West, Norway.

### Measures

The DHI is intended to measure the handicapping effects of dizziness on physical, emotional and functional sub-domains [1]. To examine validity and responsiveness of the DHI-N, the following condition specific and generic measures were included, all considered to be more or less associated with the DHI-N:

Vertigo Symptom Scale - short form (VSS-sf) is a condition specific questionnaire assessing perceived severity of vertigo symptoms during the last month by measuring frequency of dizziness, vertigo, imbalance and related autonomic symptoms (nausea, sweating, etc.) [32]. The scale includes 15 items, comprising two sub-scales indicating the relative impact of vertigo and balance (VSS-sf-V, 8 items) and autonomic/anxiety (VSS-sf-A, 7 items) on the total score [32,33]. Five ordinal response categories range from 'never' (score 0) to 'very often (most days)' (score 4), and give a total score ranging from 0 to 60, the VSS-sf-V ranges 0-32, and VSS-sf-A ranges 0-28, higher scores indicating more severe symptoms [32]. The Norwegian version of the VSS-sf used in the present study (VSS-sf-N), has demonstrated satisfactory psychometric properties [34].

COOP/WONCA is a generic assessment tool measuring perceived functional health status referring to the last two weeks. Six charts, each with one question, have five ordinal response categories: 1 is best and 5 is worst functioning. The charts include 'physical fitness' (A. *What was the hardest physical activity you could do for at least 2 minutes?*), 'feelings' (B. *How much have you been bothered by emotional problems such as feeling anxious, depressed, irritable or downhearted and sad?*), 'daily activities' (C. *How much difficulty have you had doing your usual activities or tasks, both inside and outside the house because of your physical and emotional health?*), 'social activities' (D. *Has your physical and emotional health limited your social activities with family, friends, neighbours or groups?*), 'changes in health' (E. *How would you rate your overall health now compared to 2 weeks ago?*), and 'overall health' (F. *How would you rate your health in general?*) [35]. Scores are derived from each individual chart (range 1-5), or as a sum score (range 5-25) of 5 charts (excluding chart E: changes in health) [35,36]. Satisfactory measurement properties have been reported in different patient populations [35,37,38], also in the Norwegian version [39-42].

The Disability Scale is a global self-report measure, and used to assess disability in connection with dizziness [43]. The scale does not refer to any time period. It is scored on a 6-point ordinal scale: 0 = 'no disability; negligible symptoms', 1 = 'no disability; bothersome symptoms', 2 = 'mild disability; performs usual work duties, but symptoms interfere with outside activities', 3 = 'moderate disability; symptoms disrupt performance of both usual work duties and outside activities', 4 = 'recent severe disability; on medical leave or had to change job because of symptoms', and 5 = 'long-term severe disability; unable to work for over 1 year or established permanent disability with compensation payment' [43]. The Disability Scale has shown excellent reliability in patients with peripheral vestibular disorders [44].

The Disability Scale seemed appropriate to use as an external anchor to examine discriminate ability and responsiveness to important change of the DHI-N. The categories of the Disability Scale differentiate levels of disability that appear clinically important to patients and clinicians, each category being easy to interpret and having intuitive face validity. Vocational disability caused by dizziness and vertigo is an infrequent cause of certified sickness absence, but people with long term sickness-absentees with dizziness/vertigo, have a considerable risk of obtaining disability pension in the future [45]. Therefore, the difference between and change in categories of the Disability Scale were used for discriminate purposes in the analysis.

Gait was assessed to measure functional balance, using a marked path of ten meters; six meters effective test distance with two meters at either end for acceleration and deceleration. Gait was registered during: 1) self-preferred gait speed, and 2) fast gait speed. One trial was offered before testing. Each person was then tested twice. Satisfactory reliability of preferred gait speed (meters pr. second) has been shown in different patient populations [46], as well as in patients with peripheral vestibular disorders [44].

### Procedures

Internal consistency, validity and responsiveness of the DHI-N were examined in sample 1. Following informed consent, consecutive participants in sample 1 received self-administered questionnaires, to be returned by mail prior to the appointment for interview and baseline testing. A second test including all measures was administered about 6 months later, using the same test procedure. The same physiotherapist interviewed and tested all participants on both occasions.

Internal consistency and test-retest reliability were examined in sample 2. The DHI-N was answered as part of a more extensive physiotherapy examination prior to a program of vestibular rehabilitation. The forms were completed twice, 48 hours apart: The first form was completed on location, the second returned by mail. The form was returned by 28 (88%) patients.

### Statistical analyses

Forms with missing values exceeding 7 items (30%) of the DHI-N total or exceeding 30% of the items in a DHI-N sub-domain, were excluded from analysis. Missing values in the included forms, were replaced by the mode value of the respective DHI-N sub-domain [47].

Demographics and test data were examined by descriptive statistics. Distributions of scores were examined by Q-Q plots and by comparing mean and median of the scales and subscales. As normality could be assumed, parametric statistics could be used. Differences between groups were checked by t-tests and ANOVA.

A possible floor and ceiling effect of the DHI-N was examined by descriptive statistics. According to Terwee et al. [27], a floor or ceiling effect is considered present, if more than 15% of the respondents have the lowest or highest score.

The underlying structure of the DHI-N was examined by exploratory factor analysis (EFA) following tests of sampling adequacy by Kaiser-Meyer-Olkin Measure (> 0.6) and Bartlett's test of Sphericity (< 0.05) [48,49]. Maximum likelihood parameter extraction technique and the scree plot were used to determine the numbers of factors to be retained for analysis [49]. The factor structure was identified by using the oblique rotation method (Oblimin) with delta = 0 allowing for moderate correlation [49]. Item loadings were evaluated in line with proposals from Costello and Osborne [50]: Item loadings < 0.40 suggest that an item is not sufficiently related to the other items in the factor, or indicates an additional factor to be explored; the minimum loading of an item is suggested = 0.32; and loadings ≥ 0.32 on two or more factors, indicate cross-loadings.

Internal consistency was examined by Cronbach's alpha, and a value > 0.80 was considered satisfactory [48].

To examine construct validity, scores of the DHI-N were correlated with those of condition specific and generic measures. Degree of linear relationships between variables were quantified by Pearson's correlation coefficient (r), and evaluated in line with guidelines proposed by Cohen [51]: r = 0.10 - 0.29 = small (low correlation); r = 0.30 - 0.49 = medium (moderate correlation); r = 0.50 - 1.0 = large (high correlation) [51]. To acknowledge the ordinal nature of the DHI, correlations were also explored by Spearman's rho, but as similar values of correlation coefficients were found, they are not reported. Analyses of the gait tests were based on the mean scores of two trials.

Regarding construct validity, we hypothesized that the impact of dizziness on quality of life assessed by the DHI-N with proposed physical, emotional and functional sub-domains, would show high correlation with symptoms of vertigo/imbalance and autonomic/anxiety of the VSS-sf-N, being related functional constructs. Additionally, since both measures are condition-specific and gather information by self-report, we expected that this pair of measures would demonstrate the highest association of all. We also hypothesized a high correlation between the DHI-N and the COOP WONCA sum score, also assessing related functional constructs. Since the DHI-N is condition specific and the COOP/WONCA a generic measure, we expected that the association in this pair of measures would be lower, than between the DHI-N and the VSS-sf-N. Since the perceived impact of dizziness assessed by the DHI-N seems important for how patients report on perceived levels of disability assessed by the Disability Scale, we expected a high correlation between these measures. We further hypothesized that the DHI-N and gait tests assessed similar physical constructs, because gait is influenced by dizziness, and gait is performed in many daily activities as well as in social situations. However, the DHI-N is a broader self-report measure, including a multitude of items, while gait tests are performance based and provide separate measures of gait. We therefore hypothesized a moderate and inverse correlation, i.e. higher perceived handicapping effect of dizziness was associated with fewer meters walked pr. second in preferred and fast gait.

As another indication of construct validity, the ability of the DHI-N to discriminate between groups with 'no disability' (scores 0-1) versus 'disability' (scores 2-5) according to the Disability Scale, was examined by ROC (Receiver Operating Characteristics) curve analyses. Considerations of the area under the ROC curve (AUC) followed guidelines presented by Hosmer and Lemeshow [52]: ≤ 0.5 no discrimination; 0.7 ≤ ROC < 0.8 acceptable discrimination; 0.8 ≤ ROC < 0.9 excellent discrimination; and ROC ≥ 0.9 outstanding discrimination. The best cut-off point of scores was identified, where the sum of the percentages of misclassified participants was lowest [52]. We hypothesized that the DHI-N would demonstrate acceptable discriminate ability (AUC ≥ 0.7).

Test-retest reliability was examined by intraclass correlation coefficients (ICC) [53]. All within-subject variability is assumed to be error of measurement in model ICC(1.1), while in model ICC(3.1) the effect of any systematic shift in data are not considered part of the error of measurement [54]. ICC values ≥ 0.70 are considered satisfactory [27,53]. Within-subject standard deviation (S_w_) denotes measurement error, and is expressed in the unit of the measurement tool. The difference between two measurements for the same subject is expected to be < 2.77 S_w _for 95% pairs of observations. A change must exceed this value in individual patients, called Smallest Detectable Difference (SDD_ind_), to claim a true change. The smallest detectable difference of a group of people (SDD_group_) can be calculated by dividing the SDD_ind _by vn [27,55]. Measurement error was also examined in a plot described by Bland and Altman [56]: Graphs with plots of individual differences between scale responses at test and retest were plotted against the mean change scores. In addition to SDD values, the 'limits of agreement' include the mean change in scores of the repeated measurements.

As an indication of responsiveness, validity of the DHI-N was explored by correlating the change scores with those of the VSS-sf-N, COOP/WONCA, Disability Scale, and gait tests. The hypothesized strength of correlations between change scores, were as previously defined for construct validity.

Responsiveness of the DHI-N was also examined by using an anchor-based method [27,57]. Scores on the Disability Scale were used as an external criterion for important change in the construct being measured, and its applicability was considered adequate [58], if changes in scores in the DHI-N and the Disability Scale correlated with r ≥ 0.50. Change scores of the Disability Scale were regrouped into 'improved', 'unchanged', and 'worsened'. 'Improved' was defined as reduced disability by 2 or more categories on the Disability Scale, 'unchanged' was defined as no change and ± 1 category change, and 'worsened' was defined as increased disability by 2 or more categories. The number of 'worsened' (n = 4) was too small to determine minimally important change for deteriorated, and they were therefore excluded from the analysis. Change scores of the DHI-N were explored in ROC curve analyses using this dichotomized scale of 'improved' and 'unchanged' participants as dependent variable. The AUC was used as a measure of responsiveness, and AUC > 0.70 is considered adequate [27]. Considerations of the AUC were as previously defined for discriminate ability. The minimally important change (MIC) was defined as the best cut-off point identified on the ROC curve to discriminate between 'improved' and 'unchanged' participants [57].

Due to missing data, the number of participants in some analyses differed from the total sample size. Level of significance was set at p-value ≤ 0.05. Statistical analyses were performed with SPSS version 16.0 for Windows.

## Results

### Study samples

The study included 92 participants in sample 1 at baseline, and 27 participants in sample 2; seven participants were excluded initially due to missing data on the DHI-N forms, six from sample 1 and one from sample 2. Details regarding descriptive information of the samples are given in Table 1. Similar mean age was seen in both samples, while the relative proportion of women was about 10% higher in sample 1. Duration of dizziness was longer in sample 1 than in sample 2. All the participants in sample 2, and the majority of participants in sample 1 had dizziness of vestibular origin, mainly represented by sequelae from vestibular neuronitis. Sample 1 also included participants with unknown origin of dizziness and non-vestibular dizziness, the latter mainly represented by anxiety, neck problems and sequelae of head and/or neck trauma. No significant differences were found in DHI-N scores between diagnostic groups, age groups, gender, duration of symptoms, or scores on applied measures.

**Table 1:** Description of the study samples

Characteristics	Sample 1 Community based n = 92	Sample 2 Tertiary referral centre n = 27
Female: n (%)	64 (70)	16 (59)
Age: mean (SD, min-max)	47.2 (11.46, 26-64)	47.5 (12.1, 24-73)
Duration of dizziness: mean months (SD, min-max)	58.2 (84.1, 2-418)	32 (51.5, 1-234)
Diagnostic groups:		
Vestibular dizziness, n (%)	59 (64)	27 (100)
Non-vestibular dizziness, n (%)	9 (10)	
Unknown origin, n (%)	24 (26)	

At the time of the second test, sample 1 had 72 participants. Eleven participants had withdrawn from the study, due to different reasons: total relief of symptoms (n = 4), no time to participate (n = 2), other diseases (n = 3), worsening of the condition (n = 1), or child birth (n = 1). In addition, six participants failed to keep test appointments despite several opportunities, and three DHI-N forms had missing data exceeding the predefined level.

### Floor or ceiling effects

The scores of the DHI-N ranged from 4 to 86 DHI points in sample 1, and 11% of the participants had < 20 DHI points and 1% had ≥ 80 DHI points. No floor or ceiling effects were demonstrated.

### Factor structure

**Figure 1:**
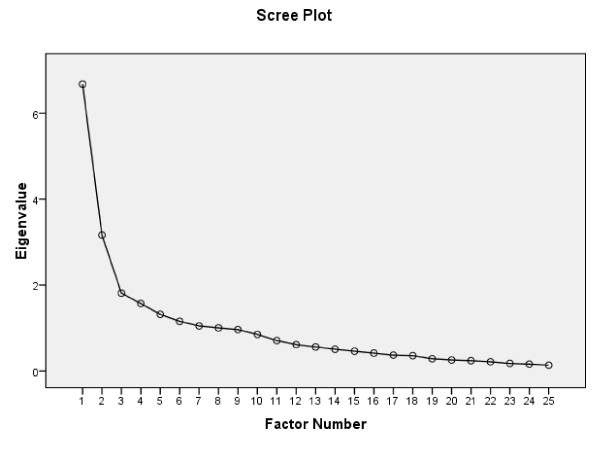
**Scree Plot of eigenvalues of DHI-N items by exploratory factor analysis (EFA) (n = 92, sample 1)**.

Exploratory factor analysis revealed eight factors in the DHI-N with eigenvalues > 1, which explained 71% of the variance before rotation. The scree plot (Figure 1) indicated two obvious factors to be retained for rotation. Factor I comprised almost all items included in the original emotional subscale, in addition to four items in the functional subscale (Table 2). Factor II comprised items included in the original physical subscale, in addition to one from the emotional and four from the functional subscales (Table 2). The two factors had low correlation (r = 0.33) with delta set at zero. Five items were below minimum loading (items 4, 10, 12, 17, and 20). Two items cross-loaded (item 16 and 22), and two items (item15 and 16) indicated a possible additional factor (Table 2).

**Table 2:** Factor structure and item loadings of the DHI-N by exploratory factor analysis (n = 92, sample 1)

Abbreviated item description^a^		DHI - Norwegian version^b ^ 2 - factor solution		DHI - Norwegian version^b ^ 3 - factor solution		
		
		Factor I	Factor II	**Factor I**.	**Factor II**.	**Factor III**.
**Physical**						
1	Looking up	- 0.15	**0.73**	- 0.28	**0.65**	- 0.25
4	Walking down aisle	0.24	0.28	0.15	0.26	- 0.17
8	Ambitious activities	0.14	**0.53**	0.13	**0.56**	- 0.02
11	Quick movements of head	< 0.01	**0.58**	- 0.09	**0.56**	- 0.06
13	Turning over in bed	< 0.01	**0.55**	- 0.08	**0.54**	0.03
17	Walking down a sidewalk	0.11	0.30	0.10	0.32	> 0.01
25	Bending over	- 0.12	**0.76**	- 0.14	**0.74**	- 0.05
**Emotional**						
2	Feel frustrated	**0.55**	< 0.01	**0.51**	0.10	- 0.04
9	Leave home alone	**0.43**	0.24	0.01	0.08	**- 0.90**
10	Embarrassed in front of others	0.18	< 0.01	0.11	0.06	- 0.10
15	Afraid people think you intoxicated	0.19	**0.35**	**0.54**	0.36	- 0.03
18	Concentrate	**0.69**	< 0.01	**0.69**	0.14	> 0.01
20	Afraid to stay home alone	0.31	< 0.01	0.03	- 0.06	**- 0.57**
21	Feel handicapped	**0.79**	- 0.14	**0.80**	- 0.05	0.02
22	Stress on relationships	**0.74**	- 0.39	**0.63**	- 0.36	- 0.20
23	Depressed	**0.61**	< 0.01	**0.50**	- 0.05	- 0.20
**Functional**						
3	Restrict travel	**0.61**	0.11	0.38	0.05	**- 0.44**
5	Getting into/out of bed	< 0.01	**0.64**	- 0.05	**0.65**	0.28
6	Social activities	**0.79**	< 0.01	**0.68**	- 0.05	- 0.21
7	Reading	0.29	**0.40**	0.39	**0.51**	0.21
12	Avoid heights	0.14	0.30	0.13	0.31	- 0.01
14	Strenuous house/yard work	**0.43**	0.29	**0.35**	0.30	- 0.17
16	Walk by yourself	0.35	**0.37**	0.05	0.28	**- 0.63**
19	Walk around in the dark	0.16	**0.52**	0.15	**0.54**	- 0.01
24	Job/household responsibilities	**0.71**	0.10	**0.80**	0.23	0.14

^a^Item loadings are presented according to the abbreviated item description of sub domains of the original version of the DHI questionnaire (physical, emotional and functional subscales). Major loadings for every item ≥ 0.32 are bold face. ^b^Exploratory factor analysis with Maximum likelihood parameter extraction method with oblique rotation (pattern matrix).

In the 3-factor solution, factor I comprised items originally included in the emotional and functional subscales (Table 2). Factor II comprised items from the original physical in addition to functional subscales. Factor III comprised two items from the original emotional and two from the functional subscales. The correlation between the three factors was low (-0.36 ≤ r ≥ 0.26) with delta set at zero. Three items loaded below minimum (items 4, 10, and 12), and four items cross-loaded (item 3, 7, 15 and 22), indicating a possible additional factor (Table 2). A four factor solution was also explored: two items cross-loaded (7, and 22), three items loaded below minimum (4, 14 and 17), and the fourth factor included only three items. Results from the EFA revealed that the items of the DHI-N loaded differently, than the suggested three sub-domains of the original version. In further analysis, only measurement properties for the total scale were thus examined.

### Internal consistency

Acceptable Cronbach's alpha values were indicated for the DHI-N in sample 1, α = 0.88, and in sample 2, α = 0.95. All items had item-total correlation > 0.20.

### Construct validity

High correlations were shown between the DHI-N and the VSS-sf-N total, the VSS-sf-N sub-scales, the COOP/WONCA and the Disability scale (r ranging 0.50 - 0.69) (Table 3). The highest correlation was found between the DHI-N and VSS-sf-N total (r = 0.69). The association with COOP/WONCA sum score was, however, almost as high (r = 0.60), the individual charts also showing moderate to high correlations (excluding chart E. *Changes in health*). Moderate correlations between DHI-N and gait tests (preferred gait: r = -0.36, and fast gait: r = -0.40) were found (Table 3).

**Table 3:** Tests, scores and examination of validity of the DHI-N (n = 92, sample 1)

Outcome measures (scale range)	Baseline Mean (SD), Range	DHI-N total Pearson's r
DHI-N total (0 - 100)	39.91 (18.95), 4 - 86	1
VSS-SF-N total (0 - 60)	14.58 (9.87), 0 - 49	0.69**
VSS-SF-V-N (0 - 32)	8.63 (6.98), 0 - 29	0.64**
VSS-SF- A-N (0 - 28)	5.95 (4.58), 0 - 20	0.50**
COOP/WONCA (5 - 25)	12.49 (3.52), 4 - 22	0.60**
A Physical (1-5)	2.48 (1.01)	0.34**
B Feelings (1-5)	2.76 (1.03)	0.35**
C Daily activities (1-5)	2.36 (0.95)	0.54**
D Social activities (1-5)	2.32 (1.09)	0.48**
E Change in health (1-5)	2.81 (0.68)	0.07
F Overall health (1-5)	2.85 (0.79)	0.43**
Disability Scale (0 - 5)	2.58 (1.29), 0 - 5	0.58**
Preferred gait (m/sec)	1.28 (0.27), 0.38 - 1.98	- 0.36**
Fast gait(m/sec)	2.00 (0.33), 1.17 - 3.00	- 0.40**

* p < 0.05 (2-tailed), ** p < 0.01 (2-tailed).

### Discriminate ability

**Figure 2:**
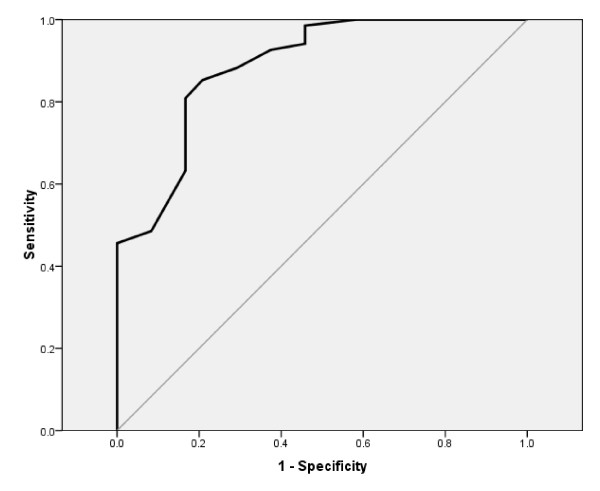
**Ability of the DHI-N to discriminate between patients with ' disability' and 'no disability' examined by ROC curve analysis (n = 92, sample 1)**.

The DHI-N showed excellent ability to discriminate between participants who reported 'disability' (n = 68) and 'no disability' (n = 24), according to the area under the ROC curve: AUC being 0.89 (95% CI 0.81-0.97), as shown in Figure 2. The cut-off point for best discrimination was 29 points, correctly classifying 85% of participants with 'disability' and 79% with 'no disability'. Those who reported 'disability' had a mean (SD) score of 46.4 (16.56) points, and those who reported 'no disability' had a mean (SD) score of 21.6 (12.13) points.

### Test-Retest reliability

Test-retest reliability of the DHI-N was satisfactory (ICC 1,1 = 0.90). Mean scores of the first test were somewhat higher than retest scores, but the difference between ICC(1,1) and ICC(3,1) analysis was minimal, showing little systematic change from the first to the second test. Absolute agreement (S_w_) was 7.1. The smallest detectable difference for an individual (SDD_ind_) was accordingly 19.67 points on the DHI-N, while the smallest detectable difference for a group (SDD_group_) was 3.78 points.

**Figure 3:**
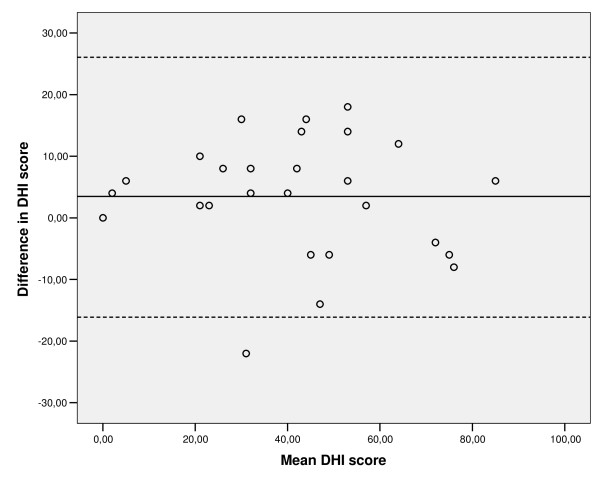
**Intra-individual differences between the DHI-N scores at test and retest plotted against the mean DHI-N change scores (n = 27, sample 2)**. The central horizontal line represents the mean difference in scores of repeated measurements, and the dotted lines represent the 95% limits of agreement.

The central line in the Bland-Altman plot (Figure 3) shows the mean change in scores from the first to the second measurement, and the flanking dotted lines, the limits of agreement, take the mean change in scores as well as the SDD_ind _into consideration.

### Responsiveness

The correlations between change in DHI-N scores and those of the other self-report measures were high, correlation coefficients (r) ranging 0.50-0.57 (Table 4). Highest association was found between change in the DHI-N and the condition specific VSS-sf-N (r = 0.57). Changes in VSS-sf-N sub-scores had similar associations with the DHI-N (VSS-sf-V-N, r = 0.51, VSS-sf-A-N, r = 0.50). The association with the generic COOP/WONCA sum score (r = 0.56) was almost as high as the VSS-sf-N total, while the change scores of each COOP/WONCA chart were moderate to high (excluding chart E. *Change in health)*. Low correlations of change scores between DHI-N and gait tests did not reach statistical significance (Table 4).

**Table 4:** Responsiveness; correlations between change scores of the DHI-N and other measures (n = 72, sample 1)

Outcome measures, change	DHI-N total, change Pearson's r
VSS - SF-N total	0.57**
VSS - SF-V-N	0.51**
VSS - SF-A-N	0.50**
COOP/WONCA (A, B, C, D, F)	0.56**
A Physical	0.30*
B Feelings	0.40**
C Daily activities	0.39**
D Social activities	0.52**
E Change in health	0.02
F Overall health	0.39**
Disability scale	0.51**
Preferred gait	0.10
Fast gait	0.20

* p < 0.05 (2-tailed), ** p < 0.01 (2-tailed).

**Figure 4:**
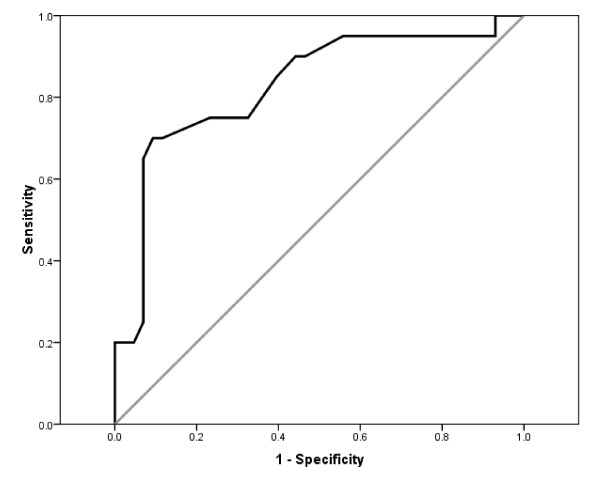
**Ability of the change scores of DHI-N to discriminate between 'improved' versus 'unchanged' participants examined by ROC curve analysis (n = 63, sample 1)**.

The Disability scale was found suitable as an external criterion of change in the construct being measured, r being 0.51 (Table 4). A significant difference in change of the DHI-N scores (<0.001) was found between the 'improved' group (n = 20) and the 'unchanged' group (n = 43) (Table 5). The scale demonstrated excellent ability to discriminate between 'improved' and 'unchanged' participants according to the area under the ROC curve: AUC being 0.83 (95% CI: 0.71-0.94), as shown in Figure 4. The anchor based MIC was identified as 11 points (Table 5), correctly classifying 75% of the 'improved' and 77% of the 'unchanged' participants.

**Table 5:** Responsiveness; ability of the DHI-N change scores to discriminate between participants reported to be 'improved' versus 'unchanged' on the Disability scale (n = 63, sample 1).

DHI-N change	Baseline scores Mean (SD)	Test 2 scores Mean (SD)	Change scores Mean (SD)	Smallest Detectable Difference	Area under the ROC curve (95% CI)	ROC cut-off point: MIC-ROC
Improved	42.70 (15.14)	24.40 (14.93)	18.30 (12.64)	19.67	0.83	11
Unchanged	38.23 (19.55)	34.05 (18.93)	4.19 (9.47)		(0.71 - 0.94)	

## Discussion

In this cross-sectional and longitudinal study of patients with dizziness, measurement properties of a translated and adapted Norwegian version of the Dizziness Handicap Inventory (DHI-N), were examined. The factor analysis revealed a different factor structure than suggested in the original version, resulting in dismissal of subscale scores. Satisfactory internal consistency of the total scale was found. Concurrent correlation between the DHI-N and other measures of related constructs were moderate to high, highest for the VSS-sf-N and lowest for preferred gait speed. The DHI-N demonstrated excellent ability to discriminate between participants with and without 'disability', AUC being 0.89, and the best cut-off point for discrimination was 29 points. Satisfactory test-retest reliability was demonstrated, and change should be ≥ 20 DHI-N points for an individual (SDD) to exceed measurement error. Correlation between change scores of the DHI-N and those of other self report measures, were high. The DHI-N demonstrated excellent ability to discriminate between self-perceived 'improved' versus 'unchanged' participants, AUC being 0.83. The anchor based MIC was identified as 11 DHI-N points. Measurement properties of the DHI-N seemed, accordingly, to be highly satisfactory.

### Translation

The items included in the DHI, were considered relevant and adequate for dizzy patients in the Norwegian culture, which was a prerequisite for translating the measure [29]. Recommended guidelines were followed [28,29], and as all the steps in the translation process were reported, the process can be validated by others [30]. The response categories and scoring system were initially kept in line with the original suggestions ('yes', 'no', 'sometimes') [1], and as reported in a previous publication [59]. However, to be in line with a recently published version [31], the sequence of response categories were changed, as shown in Additional file 1. A one page or a two page questionnaire would be favourable to eliminate the problem of missing data from unanswered backside pages.

### Study samples

As recommended when developing an assessment tool for a particular population [27], the recruitment of dizzy patients was broad, with participants from primary health care, as well as from tertiary referral centres, settings in which the DHI-N questionnaire will be used in the future. The mean age and gender of the participants in sample 2, were comparable to the participants included when the original DHI scale was developed and tested [1]. The target population of the DHI was patients with vestibular system disease, and it might be argued that the DHI, therefore, should not be used in patients with dizziness of other origins. Sample 1 had a broader recruitment, and also included participants with non vestibular and unknown origin of dizziness, and was thus neither directly comparable to sample 2, nor to the sample used in development of the scale. However, patients seen at tertiary referral centres are referred from General Practitioners in primary health care and from other medical specialists. The reason for referral is often associated with uncertain aetiologies, thus probably presenting a multitude of origins. Therefore, dizziness, rather than the origin of dizziness, should probably be the indication for using the questionnaire. It was favourable that the participants in the present study reported a wide range of scores on the DHI-N questionnaire, without showing floor or ceiling effects. In that way, measurement properties of the broad scale scores have been taken into consideration.

In our study, the sample sizes for testing measurement properties of the DHI-N, seem mostly adequate, according to quality criteria proposed by Terwee et al. [27]. A sample size ≥ 50 is, however, proposed in test-retest reliability studies [27], while in our study of test-retest reliability, only 27 participants were included. SDD_ind _estimated in sample 2 were in line with the initial findings in the DHI (SDD ≥ 18) [1]. However, previous studies with larger sample sizes, have demonstrated a smaller SDD_ind _in the DHI [8,16]. Our results are at least safe estimates of measurement error, but later studies of reliability should preferably include a larger sample size. The sample size for the factor analysis should preferably be 4-10 subjects pr item [27,50]. However, as acceptable sampling adequacy was demonstrated, we considered the sample size (n = 92) acceptable for exploring the factor structure in the present study.

### Factor structure and internal consistency

We applied exploratory factor analysis (EFA), which is recommended when the factor structure of a measure has not been established [49,50]. The analysis did not confirm the originally suggested content domains of the DHI. Previous results from principal components analysis (PCA) of the DHI in the original language [9], as well as of other translated versions [10,11], have demonstrated various underlying factor structures. Different results from factor analyses of the same instrument may have several causes, such as use of different methods of analyses (EFA versus PCA), translation, patient samples, and sample size, but might also indicate limitations in item construction, and that the initial factor structure could be flawed [29,50]. According to a recent publication, the authors of the original version have also abandoned calculations of subscale scores [31]. Internal consistency of the DHI-N total scale by Cronbach's alpha was above the recommended limits [48], and in line with previous results [1].

### Construct validity

Construct validity of the DHI-N was supported, as the predefined hypotheses of concurrent correlations with other measures, were confirmed. The high and highest correlation was demonstrated between the DHI-N and the VSS-sf-N, and was also high for the VSS-sf-N subscale scores. Although the DHI-N subscale scores were abandoned in the present study, the results indicate that the DHI-N includes similar physical and emotional constructs, as the VSS-sf-N. Using the concepts from International Classification of Function (ICF) [60], these condition specific questionnaires appear to measure similar constructs, but at different functional levels. While the DHI-N items appear to capture the limiting effect of dizziness on performance of activities, the VSS-sf-N items appear to capture severity of symptoms, reflecting impairments of body functions [60].

The hypothesis of high association between the sum scores of the DHI-N and COOP/WONCA was confirmed, but the association was higher than expected, taking into consideration that the COOP/WONCA is a generic measure. The high association indicates similarity of functional constructs. The handicapping effect of dizziness (DHI-N) and functional health status (COOP/WONCA sum score) may represent related functional problems according to ICF [60]. Both ask questions about performance of activities and/or limitations and participation in different areas of every-day life. The association was found to be particularly high between DHI-N total score and the COOP/WONCA chart C. *daily activities*. Previous findings of correlations between the DHI and subscales of the generic SF-36, ranged from 0.11 to 0.71 [15,16]: Fielder et al. [15] found high associations between the DHI total score and 8 sub scores of the SF-36 (Spearman's rho ≥ 0.53), while the findings of Enloe and Shields [16] showed variable associations with the DHI sub-scores. Results from the present and the previous studies, indicate associations between two versions of the DHI and two generic measures of health.

The hypothesis of moderate correlation between the DHI-N and gait as a measure of balance was also confirmed. We might have expected even higher correlation, since patients with dizziness tend to have impaired balance. However, taking into consideration that the DHI-N is a broad self-report measure and gait tests a performance measure that only yields one test result, a moderate correlation is more realistic [48]. The results from participants with multiple origins of dizziness, are thus also in line with previous findings from patients with vestibular disorders [13,61]. The results support construct validity of the DHI-N.

In agreement with several authors [27,46,48,62], the ability to discriminate between participant groups that are known to have a trait or condition of interest, and those who do not (i.e. discriminate between 'known' groups), was used to indicate construct validity of the DHI-N. The questionnaire was shown to have excellent ability to discriminate between dizzy patients with and without perceived 'disability', according to the Disability Scale. The hypothesis of acceptable discrimination was confirmed, and construct validity was supported. The optimal cut-off point found in this study also corresponds to previous findings of 'mild' self perceived handicap, ranging 0-30 points on the DHI [13]. Previously, the DHI has also shown ability to discriminate between groups of dizzy patients according to frequency of dizziness episodes [1], and levels of functional impairment [13].

### Test-retest reliability

Relative test-retest reliability was satisfactory [53], and comparable to initial results by Jacobson and Newman [1]. The risk of recall bias in the present study was considered minimal, since filling out the form was part of an extensive test battery and separated by 48 hours. A somewhat higher correlation seen in the original study may be due to short retest interval (same day). There are no definite guidelines as to how long the time interval should be, however, time should be long enough to secure that previous self-reported responses are forgotten, and short enough for stability of the condition to be retained [48].

Knowledge of absolute reliability of an instrument allows identification of change beyond measurement error. No absolute value is recommended, but should preferably be small for instruments to be useful as an outcome measure. The SDD for an individual in the present study was somewhat large (20 DHI-N points), but is similar to the value reported in the original study (18 points) [1]. The SDD_ind _makes it possible to judge whether or not a change is above measurement error, as recommended by Terwee et al [27]. As there tended to be a systematic change in scores between repeated measurements (Figure 3), this should probably also be taken into consideration when judging change scores.

### Responsiveness

Responsiveness of the DHI-N was supported, as the hypotheses of correlations between change scores of the DHI-N versus the VSS-sf-N total, as well as the COOP/WONCA sum, were confirmed. However, the hypotheses of correlations with performance based gait tests were not confirmed. The highest association between change scores of the DHI-N and the VSS-sf-N indicated similar constructs; a reduction in perceived handicapping effect of dizziness was associated with a reduction in perceived frequency of symptoms of dizziness. The correlation with change scores of the COOP/WONCA sum was shown to be almost as high; reduction in the perceived handicapping effects of dizziness was associated with better functional health. This is in line with the associations that were found between the scales in cross-sectional analysis.

The scale did not show significant relationship with changes in gait speed. Previously, moderate correlation between change in DHI and change in the mean score of equilibrium, derived from six sensory conditions assessed by Computerized Dynamic Posturography (CDP), has been demonstrated [63]. According to Finch et al. [46], change scores of measures at different functional levels (ICF) could be expected to correlate between r = 0.2 - 0.5. The lack of relationship with gait tests in the present study, may imply that although gait speed is considered a measure of functional balance and disability, gait is perhaps more a physical characteristic, than a construct [46]. The use of change in gait speed to validate change in the DHI-N scale may therefore be questioned.

Responsiveness was further supported by the ability of the DHI-N to discriminate between self-perceived clinically 'improved' and 'unchanged' participants. The criterion for improvement was a reduction of 2 or more categories on the Disability Scale. The applicability of the Disability Scale as external criterion of important change, was found acceptable according to a review of current approaches to defining clinically meaningful change [58], although, according to criteria suggested by Terwee et al [27], a stronger correlation is preferable. The content of the DHI was, however, designed to capture several aspects of self-perceived consequences of dizziness that no previous questionnaires had covered, thus there is no 'gold standard'. The Disability Scale assesses self-perceived disability, has favourable levels of ordinal categories, a change in categories imply important clinical change, and high concurrent correlation with the DHI-N indicates similar functional constructs. The same measures were used at baseline and follow-up, reducing possible biases that are reported from use of scales, where the client must estimate change from a previous state at an earlier time [48].

The area under the ROC curve indicated excellent discriminate ability according to recommended limits [27]. However, the optimal cut-off point of 11 DHI-N points, the anchor based MIC, was within the limits of measurement error at the level of an individual (SDD_ind _≥ 20 DHI-N points), but exceeded the level estimated for groups (SDD_group _≥ 4 DHI-N points). Thus, the DHI-N is considered responsive in the construct being measured, but a real change must exceed measurement error.

Ability to measure change in an instrument have been examined by different methods [26,55,64-66]. Several authorities [55,64,66], define sensitivity to change as the ability of an instrument to detect change in general, while responsiveness is defined as the ability of an instrument to detect a clinically important change, and a real change in the concept being measured. The DHI has been used in previous studies to explore change in general and change in scores due to effect of treatment [7,12,16], thus indicating sensitivity of the DHI, according to the definitions above. The ability of the DHI to discriminate between change scores in groups of participants with dizziness who were expected to change differently according to the treatment received, has been demonstrated in the original version [18,21,23,24], and also in a translated version [17,25]. These studies did, however, not address responsiveness as a quality of the DHI questionnaire to detect important and real change in the constructs being measured. The present study is the first to address and demonstrate this ability in the DHI scale, i.e. to detect self-perceived important change in the construct being measured using an anchor based approach.

### Challenges and limitations

Several challenges and limitations of the present study have already been discussed, also in relation to quality criteria for measurement properties proposed by Terwee et al. [27]. We recognize that the widely used DHI has limitations in itself, having only three response categories for each item to describe the handicapping effect of dizziness, and to capture change. It is a challenge that the subscales are used in the original DHI, while we recommend that only the sum scale should be used, since this relates to the question of equivalence between the scales. The use of the Disability Scale as an anchor for important change in the DHI-N seems appropriate, since it reflects important levels of functioning for the individual. Other relevant external criteria of important change might also be explored in future studies, still realizing the lack of 'a golden standard'.

## Conclusions

The total scale of the Dizziness Handicap Inventory, Norwegian version demonstrated satisfactory measurement properties as a discriminate and evaluative measure, and can therefore be used to assess the impact of dizziness on quality of life in Norwegian speaking patients. This is the first study that has addressed and demonstrated anchor based responsiveness of the DHI to self-perceived clinically important change, also providing values of SDD, and MIC to help interpret change scores.

## List of abbreviations

AUC: Area under the ROC curve; DHI: Dizziness Handicap Inventory; DHI-N: Dizziness Handicap Inventory, Norwegian version; EF: Exploratory factor analysis; MIC: Minimally important change; PCA: Principal component analysis; ROC: Receiver operating characteristic; SDD: Smallest detectable difference; VSS-sf: Vertigo Symptom Scale - short form; VSS-sf-N: Vertigo Symptom Scale - short form - Norwegian version; VSS-sf-V: Vertigo Symptom Scale - short form - vertigo/balance subscale; VSS-sf-V-N: Vertigo Symptom Scale - short form- vertigo/balance subscale - Norwegian version; VSS-sf-A: Vertigo Symptom Scale - short form- autonomic/anxiety subscale; VSS-sf-A-N: Vertigo Symptom Scale - short form - autonomic/anxiety subscale - Norwegian version.

## Competing interests

The authors declare that they have no competing interests.

## Authors' contributions

A-LT designed and carried out the study using sample 1, performed the statistical analysis of data from sample 1: factor analysis, internal consistency, validity, discriminate ability and responsiveness, drafted and wrote the article. KTW designed and carried out the test-retest study using sample 2, performed statistical analysis of data in test-retest reliability and internal consistency, helped to interpret results, to draft and write the article. LIS contributed to plan the article and relevant statistical analysis, helped to interpret results, to draft and write the article. All authors read and approved the final version.

## Supplementary Material

Additional file 1 **Dizziness Handicap Inventory - Norwegian version (DHI-N)**. Questionnaire (Norwegian version) of the Dizziness Handicap Inventory (DHI-N). Click here for file
