# Supplementary material for: Measurement properties of the Dizziness Handicap Inventory by cross-sectional and longitudinal designs
Source: Health Qual Life Outcomes. 2009 Dec 21;7:101. doi: 10.1186/1477-7525-7-101 (PMC2804706; doi:10.1186/1477-7525-7-101)
Supplement: Additional file 1 — Dizziness Handicap Inventory - Norwegian version (DHI-N). Questionnaire (Norwegian version) of the Dizziness Handicap Inventory (DHI-N). [file 1477-7525-7-101-S1.DOC]

# Additional files

## Additional file 1 - Dizziness Handicap Inventory - Norwegian version (DHI-N)

| Instruksjon: Hensikten med dette skjemaet er å identifisere vanskeligheter du kan oppleve på grunn av din svimmelhet eller ustøhet. Vennligst besvar hvert av spørsmålene med ‘ja’, ‘nei’ eller ‘noen ganger’. **Besvar hvert spørsmål ut fra at det bare er forbundet med ditt svimmelhets- eller ustøhetsproblem.** | | | |
| --- | --- | --- | --- |
|  | Ja  (4) | Noen ganger  (2) | Nei  (0) |
| 1. Øker problemet ditt når du ser opp? |  |  |  |
| 2. Føler du deg frustrert på grunn av problemet ditt? |  |  |  |
| 3. Begrenser du reising i jobb eller fritid på grunn av problemet ditt? |  |  |  |
| 4. Øker problemet ditt når du går mellom reolene i et supermarked? |  |  |  |
| 5. Har du vansker med å komme deg inn eller ut av seng på grunn av problemet ditt? |  |  |  |
| 6. Hemmer ditt problem deg i betydelig grad fra å delta i sosiale aktiviteter som å gå ut på middag, kino, dans eller i selskap? |  |  |  |
| 7. Har du vansker med å lese på grunn av problemet ditt? |  |  |  |
| 8. Øker problemet ditt når du utfører mer ambisiøse aktiviteter som sport, dans og husarbeid som å feie gulv eller sette oppvasken på plass? |  |  |  |
| 9. Er du redd for å gå hjemmefra uten å ha noen til å følge deg på grunn av problemet ditt? |  |  |  |
| 10. Har du vært forlegen/flau foran andre på grunn av problemet ditt? |  |  |  |
| 11. Øker problemet ditt når du snur fort på hode? |  |  |  |
| 12. Unngår du høyder på grunn av problemet ditt? |  |  |  |
| 13. Øker problemet ditt når du snur deg i sengen? |  |  |  |
| 14. Er det vanskelig for deg å utføre anstrengende husarbeid eller hagearbeid på grunn av problemet ditt? |  |  |  |
| 15. På grunn av problemet ditt er du redd for at folk kan tro at du er (be)ruset? |  |  |  |
| 16. Er det vanskelig for deg å gå på en tur alene på grunn av problemet ditt? |  |  |  |
| 17. Øker problemet ditt når du går langs et fortau? |  |  |  |
| 18. Er det vanskelig for deg å konsentrere deg på grunn av problemet ditt? |  |  |  |
| 19. Er det vanskelig for deg å gå rundt i huset ditt i mørket på grunn av problemet ditt? |  |  |  |
| 20. Er du redd for å være alene hjemme på grunn av problemet ditt? |  |  |  |
| 21. Føler du deg handikappet på grunn av problemet ditt? |  |  |  |
| 22. Har problemet ditt vært belastende på ditt forhold til familiemedlemmer eller venner? |  |  |  |
| 23. Er du deprimert på grunn av problemet ditt? |  |  |  |
| 24. Forstyrrer problemet ditt deg i å ivareta dine forpliktelser i jobb eller hjemme? |  |  |  |
| 25. Øker dine problemer når du bøyer deg forover? |  |  |  |
| TOTAL (0-100 poeng) |  |  |  |
